# Effects of a Web-Based, Evolutionary Mismatch-Framed Intervention Targeting Physical Activity and Diet: a Randomised Controlled Trial

**DOI:** 10.1007/s12529-019-09821-3

**Published:** 2019-10-25

**Authors:** Elisabeth B. Grey, Dylan Thompson, Fiona B. Gillison

**Affiliations:** 1 Centre for Motivation and Health Behaviour Change, Department for Health, University of Bath, Bath, BA2 7AY UK; 2 Department for Health, University of Bath, Bath, BA2 7AY UK

**Keywords:** Behaviour change, Physical activity, Diet, Randomised controlled trial, Web-based intervention

## Abstract

**Background:**

This study sought to test the effectiveness of a 12-week, novel online intervention (Evolife) aiming to increase physical activity level (PAL) and reduce energy intake (EI) among overweight/obese adults. The intervention used an evolutionary mismatch message to frame health information in an engaging way, incorporating evidence-based behaviour change techniques to promote autonomous motivation, self-efficacy and self-regulatory skills.

**Method:**

Men and women aged 35–74 years with a BMI of 25–40 kg/m^2^ were eligible. Participants were randomised to receive either the intervention (comprising a face-to-face introductory session, 12 weeks’ access to the Evolife website and a pedometer) or a control condition (face-to-face introductory session and NHS online health resources). PAL was measured objectively and EI was self-reported using 3-day weighed food records. Secondary measures included BMI, waist circumference and blood pressure.

**Results:**

Sixty people met inclusion criteria; 59 (30 intervention) completed the trial (mean age = 50; 56% male). Differences between groups’ change scores for PAL and EI were of small effect size but did not reach significance (d = 0.32 and d = − 0.49, respectively). Improvements were found in both groups for PAL (int: d = 0.33; control: d = 0.04), EI (int: d = − 0.81; control: d = − 0.16), waist circumference (int: d = − 0.30; control: d = − 0.17) and systolic blood pressure (int: d = − 0.67; control: d = − 0.28).

**Conclusion:**

The intervention did not lead to significantly greater improvement in PAL or reduction in EI than a minimal intervention control, although the changes in the intervention group were of meaningful effect size and comparable with positive outcomes in larger intervention trials.

**Trial Registration:**

This trail was registered on www.clinicaltrials.gov on 16 January 2017 (appeared online 26 January 2017), reference NCT03032731.

## Introduction

Sedentary lifestyles and poor dietary behaviours are major contributors to obesity and associated non-communicable chronic diseases, such as type 2 diabetes and cardiovascular disorder [1]. Unfortunately, physical inactivity and poor diets are becoming increasingly prevalent in many nations, leading to high health and social costs; in the UK alone, it has been predicted that by 2050, the population obesity rate could be 40%, with associated annual costs of £49.9 billion [2]. Improving people’s physical activity and dietary intake is thus recognised as a public health priority [3].

Given the scale of the problem, interventions to change activity and dietary behaviours need to reach large populations [4]. While many health behaviour change interventions have been developed, most are delivered via a series of face-to-face sessions, which are expensive and limits reach [5]. Increasingly, researchers have been investigating using the Internet to deliver health interventions on computers and mobile devices, which has the potential to reach large audiences. The Internet is well-placed to deliver self-directed interventions (i.e. those requiring no professional contact or simply an introductory face-to-face session [6]). If effective, self-directed interventions present a low-cost means of promoting healthy individual lifestyle change, requiring no or relatively less face-to-face time with specialist personnel [7]. Reviews have indicated that Internet-based interventions, both self-directed and supported by face-to-face sessions, can lead to significant improvement in both physical activity and diet, at least in the short term [8–11]. However, these reviews have highlighted that more research is needed on which behaviour change techniques to include and how best to maximise user engagement.

The present study sought to explore the effectiveness of an Internet-based, self-directed intervention aiming to promote increases in physical activity level (PAL) and reductions in energy intake (EI) among overweight and obese men and women aged 35 to 74 years. This population is at increased risk of developing type 2 diabetes and cardiovascular disorders, and there is a need for effective interventions to improve their modifiable lifestyle behaviours [12–14]. The intervention was developed by the research team and used the concept of an evolutionary mismatch to frame health information about physical activity and diet in a novel and engaging way. The evolutionary mismatch concept proposes that changes to the human cultural environment have occurred too rapidly for genetic evolution to keep up, resulting in a mismatch between our genes and environment that leads to chronic disease [15, 16]. In particular, the human body has evolved to need much higher levels of physical activity than the modern lifestyle provides and is ill suited to process the high amounts of energy-dense foods that are common in the modern diet. Previous research found that using the mismatch concept to frame health information helped to stimulate participants’ interest in health and lifestyle advice [17]. Experimental studies have demonstrated that stimulating users’ interest in an intervention website positively predicts both intention to visit the website and the number of pages viewed when on the website [18]. The mismatch perspective also seemed to provide a rationale for making health behaviour changes: it not only helped explain *what* lifestyle factors cause disease (which is commonly covered in existing health promotion information) but also *why* these factors cause disease [17]. The present intervention differed from standard health behaviour change interventions by incorporating the evolutionary mismatch concept in order to promote user engagement with the intervention website content, which is associated with greater effectiveness of interventions [19].

The intervention is primarily delivered through a website but also comprises a single, face-to-face introductory session with a researcher for each participant, provision of pedometers and one-off, individual feedback on participants’ baseline diets. As well as providing mismatch-framed information on physical activity and diet, and advice on making behaviour changes, the intervention incorporates active behaviour change techniques (i.e. those requiring input from the user rather than the user passively receiving intervention content). Specifically, the intervention website provides an interactive platform for users to develop three self-regulatory skills (action planning, coping planning and self-monitoring) that have shown particularly good efficacy in dietary and physical activity interventions [20–22]. Reviews have indicated that Internet-based interventions incorporating elements to increase self-regulatory skills (e.g. a facility to record goals and log progress) tend to be more effective [8, 11].

The primary aim of this study was to assess the effectiveness of the 12-week evolutionary mismatch-framed, self-directed intervention in increasing PAL and reducing EI. We also examined whether any changes in activity or diet achieved by the intervention were sufficient to generate clinically meaningful changes in metabolic control and/or anthropometric risk markers for developing type 2 diabetes and cardiovascular disorder.

## Methods

### Design

A randomised controlled trial (RCT) was conducted with two parallel groups. After initial screening, participants were allocated to receive either information about freely available NHS website resources in a one-off face-to-face session with a researcher (control group) or the study intervention, using a minimisation calculation to balance the groups for age, gender and BMI [23]. Physical activity, dietary, health and psychosocial variables were assessed at baseline, 6, and 12 weeks. A CONSORT checklist for this study is provided in Additional file 1.

### Participants

Men and women aged between 35 and 74 years (inclusive) and with a body mass index (BMI) of at least 25 kg/m^2^ but less than 40 kg/m^2^ were eligible to take part. The study was advertised in local media and via social media.

As the intervention focus was on prevention, individuals were excluded if they had been diagnosed with coronary heart disease, chronic kidney disease, type 1 or type 2 diabetes, stroke, heart failure, severe hypertension (BP > 180/110 mmHg), peripheral arterial disease or thyroid disorders. In order to assess the effects of the intervention in isolation, individuals were also excluded if they were currently taking any medications that could affect their weight, were going through the menopause, were taking part (or had participated within the last 2 months) in another lifestyle intervention or had recently undergone a large change in habitual lifestyle or body mass. Participants had to be fluent in English and have access to the Internet.

### Sample Size

A sample size of 54, with 27 participants in each group, was calculated as the minimum required to detect a between-group difference in change in physical activity level (PAL; defined as total daily energy expenditure divided by basal metabolic rate); this calculation used a predicted effect size of 0.69 (based on the results of a similar 12-week intervention, which informed participants of the effects of physical activity and supported them to monitor and set goals to increase their activity behaviour, conducted with a similar population of inactive, overweight adults [24]) with power of 0.80 and alpha of 0.05. To allow for drop-outs, a recruitment target of 60 participants was set.

### Procedure

Individuals who contacted the researchers to express an interest in the study were screened via e-mail or telephone, and those meeting eligibility criteria were sent an information document. Those who met the eligibility criteria and remained interested in participating after reading the information sheet were given the opportunity to ask the researcher any questions about the study before being sent a consent form to complete. When informed consent had been received, a baseline assessment appointment was scheduled.

#### Assessments

All assessment visits took place at a laboratory in the University with a researcher. Participants were asked to arrive in the morning, in a fasted state (having consumed nothing except water since 10 p.m. the day before). On arriving at the lab, participants were asked to complete a questionnaire pack, which took between 15 and 25 min, and then blood pressure was recorded while the participant was still seated. Next, height, weight and waist circumference were measured before taking a 10-ml blood sample. Finally, the researcher gave participants a multisensory physical activity monitor to wear for 7 days following the assessment visit, along with a 3-day food diary and set of kitchen scales to also complete over the following week. Full instructions on both the activity monitor and food diary were explained by the researcher and given in printed form for the participants to take away. Activity monitors and completed food diaries were collected by the researcher approximately 8 days after each assessment (i.e. when the activity monitoring had been completed).

#### Allocation

Following the baseline assessment visit, participants were allocated to one of the two groups using a concealed minimisation procedure, which dynamically adjusts allocation probabilities in order to minimise differences between groups on important covariates [23, 25]. A researcher in the university (who otherwise was not involved with the study in order to limit bias [23]) entered the participants’ age, gender and BMI values into an Excel database programmed to calculate group allocations such that differences in age, gender and BMI between groups would be minimised [adapted from 26]. Participants were informed to which group they had been allocated.

### Intervention

The intervention (“Evolife”) was based around a website that aimed to provide participants with information, framed from an evolutionary mismatch perspective, about physical activity and healthy eating, and advice on how to make behavioural changes to improve health. The mismatch concept was incorporated in the website text (providing an overview of the concept and using it to frame health information from an evolutionary perspective), and graphics were used to convey the mismatch and health concepts in engaging, quickly understandable ways (e.g. an interactive timeline displaying key milestones in evolution and the impacts these had on activity levels and diet). Graphic designers and web developers were employed to create an attractive, professional-looking and user-friendly website, thus promoting engagement. The website content was developed iteratively, through a series of qualitative and quantitative pilot work. Emphasis was placed on making small behaviour changes that could gradually be increased over time to lead to a sustained healthy habit, in line with current UK guidance [7, 27]. The website also provided a personalised, interactive area for participants to set activity- and diet-related goals and plans and monitor their progress towards these. One of the goals was a daily step goal and to help with this, participants were given a pedometer. Three other goals could be set and participants were free to choose what these could be, rather than having to choose from pre-specified goals that may not have been relevant or appropriate for participants’ individual circumstances. Participants could use the three ‘free’ goals for either physical activity or diet-related behaviours. The information on the website highlighted the different forms of physical activity (e.g. moderate cardiovascular activities, strengthening activities); on the basis that any increase in physical activity would be beneficial, no particular form of activity was encouraged over others. In order to help set diet-related goals, participants were asked to complete a short food frequency questionnaire (FFQ), adapted from the British Heart Foundation’s ‘How healthy is your diet?’ questionnaire; the researcher compiled brief feedback based on the responses and e-mailed this to participants shortly after the introductory session. The feedback highlighted both aspects of the participant’s diet that met recommendations and aspects that could be improved, along with suggestions of changes that they could try. A mobile-friendly version of the website was also created to enable participants to use the website via tablets and mobile phones. The behaviour change techniques included in the intervention are displayed in Table 1. A TIDieR checklist of intervention components is provided in Additional file 2, and a more detailed description of the intervention content (including screenshots from a sample of pages from the Evolife website) is provided in Additional file 3. Intervention group participants had a one-to-one meeting after their baseline assessment with a researcher, who had a background in health psychology and behaviour change. Meetings were held either at the university or a public space convenient to the participant. The researcher provided an introduction to the intervention, giving an overview of the evolutionary mismatch concept and explaining what the intervention aimed to help them achieve. The researcher showed the participant the various areas and features of the website, particularly focusing on the goal setting and recording area while encouraging participants to explore the more informational pages in their own time. The researcher discussed with participants what types of goals, besides the step goal, they might like to set, providing examples, asking about daily routines and encouraging participants to set goals that would provide an element of challenge while also considering potential barriers. Participants were advised to read the information on the website about goal setting, planning and social support before setting their goals. Participants were also shown how to use the pedometer and allowed to ask any questions they had about the intervention, as well as completing the FFQ. To help standardise the meetings, the researcher followed a schedule and, when responding to questions, gave the same information as could be found on the website.

**Table 1:** Behaviour change techniques included in the Evolife intervention

BCTs (Taxonomy v.1, 2013)	Delivery
Goal setting (behaviour)	Face-to-face in introductory session with researcher and online
Action planning	Face-to-face in introductory session with researcher and online
Problem solving (coping planning)	Face-to-face in introductory session with researcher and online
Prompt mental rehearsal of successful performance	Face-to-face in introductory session with researcher and online
Feedback on behaviour	Face-to-face in introductory session with researcher, email and pedometer feedback and online
Prompt habit formation	Face-to-face in introductory session with researcher and online
Information about health consequences	Online
Information about antecedents	Online
Demonstration of the behaviour	Online
Social support (practical and emotional)	Online
Prompt restructuring the social environment	Online
Self-monitoring of behaviour	Pedometer and online

### Control Group

Participants in the control group had a one-to-one meeting with a researcher after their baseline assessment to be shown three freely available NHS websites for healthy living: NHS Choices Live Well www.nhs.uk/live-well, One You www.nhs.uk/oneyou and Change 4 Life www.nhs.uk/change4life. These websites aim to promote healthy lifestyles and include advice and downloadable mobile apps about physical activity, diet, alcohol and tobacco smoking. In the meeting, the researcher specifically focused on the website areas relating to physical activity and diet. The meetings lasted approximately 20 min, and then no further contact was made between the researcher and control participants except for the assessment visits. After the collection of all 12-week data, control participants were offered the intervention.

### Measures

#### Primary Outcomes

The primary outcomes were mean daily physical activity level (PAL) and mean daily energy intake (EI: measured in kilocalories, kcal). Physical activity was measured using BodyMedia SenseWear ‘Core’ monitors (BodyMedia Inc., USA). The BodyMedia armband has been shown to provide valid and reliable measures of physical activity energy expenditure [28–30]. Data from the monitors was processed using SenseWear Professional version 8 software. Participants were asked to wear the monitor for seven complete days (midnight to midnight), only removing it for water-based activities (e.g. showering). A minimum of five valid days (including both a Saturday *and* Sunday) of data was required; this is the minimum amount needed to gain a reliable measure of habitual physical activity [31]. A valid day was one in which there was data for at least 80% of a 16-h waking period. Gaps in the data (e.g. when the monitor was removed for showering) were replaced with estimated basal metabolic rate (BMR), calculated using the age- and gender-specific Schofield equation [32]. The total energy expenditure (TEE) data collected by the monitors was used to calculate PAL (PAL = TEE/BMR). The data recorded by the monitors also enabled calculation of the time participants spent in various energy expenditure thresholds [31]: sedentary = MET < 1.8, light intensity = 1.8 ≤ MET < 3, moderate intensity = 3 ≤ MET < 6, vigorous intensity = 6 ≤ MET < 10.2 and total moderate and vigorous activity, MVPA ≥ 3MET (where MET = metabolic equivalent). The activity monitors also recorded step counts.

Energy intake was measured using 3-day weighed food and fluid records, incorporating two weekdays and one weekend day, completed during the weeks that participants were wearing activity monitors. Participants were given a set of digital scales (Model ‘Disc 1036’, Salter, Kent, UK) and a diary in which to record everything they ate and drank on monitoring days. Nutritics online software (version: 4.315 Education, United Kingdom) was used to calculate nutritional content.

#### Secondary Outcomes

Blood pressure was measured using a digital sphygmomanometer (Model: EW3106, Panasonic, UK & Ireland). Waist circumference was measured with a non-stretch measuring tape (Model: 201, Seca, UK), placed approximately mid-way between the lowest rib and the iliac crest, while participants were standing and had completed a gentle exhalation [33]. For both blood pressure and waist circumference, three readings were taken and the mean value calculated. Height was measured to the nearest millimetre using a wall-mounted stadiometer (Seca, UK), participants first removed their shoes. Participants remained barefoot and were also asked to remove jackets and all items from their pockets before being weighed on digital scales (Model: BC-543, Tanita, Amsterdam, the Netherlands). Blood samples were taken by venepuncture: a 21G needle (BD Valu-Set, Becton Dickenson & Co., Plymouth, UK) was inserted into an antecubital forearm vein and a syringe (BD Valu-Set, Becton Dickenson & Co., Madrid, Spain) used to draw a 10-ml sample. Blood was processed and stored as serum or plasma using standard methods. Analysis was completed in batches with each participant’s samples from all three time points being analysed in the same batch. Total cholesterol, HDL cholesterol, triglycerides, glucose and high-sensitivity C-reactive protein (CRP—a marker of inflammation) concentrations were measured from plasma using assay kits (Randox Laboratories, Crumlin, NI) in a Daytona automated analyser following manufacturer guidelines (Rx Series, Randox Laboratories, Crumlin, NI). Insulin was measured in serum by enzyme-linked immunosorbent assay (ELISA; Mercodia, Sweden) according to manufacturer instructions.

#### Demographic Data

Questionnaires were administered at baseline to collect the following data: age, sex, ethnicity, level of education, smoking status, area deprivation (Index of Multiple Deprivation derived from postcodes), family history of cardiovascular disease or type 2 diabetes and perceived health status (using the EQ-5D-3L [34]).

#### Process Measures

Questionnaires were used to evaluate whether the intervention had an effect on the predicted underlying cognitive and affective processes of behaviour change. These evaluations were completed by all participants at baseline, 6 and 12 weeks, to assess motivation, self-efficacy, self-monitoring and action and coping planning. The BREQ-3 [35, 36] was adapted to assess motivation for physical activity and healthy eating. Self-efficacy was measured using the BARSE [37], adapted for physical activity and Pawlak and Colby’s [38] self-efficacy scale for eating a healthy diet. Adapted versions of the nine-item instrument developed by Sniehotta and colleagues [39] were used to assess action and coping planning. Participants were asked to rate on a 4-point scale the degree to which they agreed or disagreed that they engaged in various forms of planning. For physical activity, four items assessed whether participants had made action plans concerning when, where, how and how often to be physically active. For diet, three items assessed whether participants had made action plans concerning what to eat, what unhealthy foods to restrict and what healthy foods to include. To reduce participant burden, as has been done elsewhere [40], only three of the original five items were used for coping planning for both physical activity and diet (‘identifying good opportunities for action’ and ‘acting in line with intentions’ were removed as they were deemed further from the coping planning construct than the other items). A five-item instrument developed by Gillison and colleagues [40] was used to assess self-monitoring of physical activity and healthy eating over the last month. On this instrument, participants were asked to rate on a 4-point scale the degree to which they agree or disagree with statements such as ‘I have consistently monitored what I eat and how healthy it is’. Semi-structured interviews with intervention group participants were also conducted after the 12-week assessment visit, to assess participants’ experience of the intervention and identify ways that it could be improved. The findings from these interviews will be reported elsewhere, with a full process evaluation.

### Analysis

Data were analysed using IBM SPSS software version 22 [41]. Baseline characteristics were compared between groups using independent *t* tests for continuous data and chi-squared tests for categorical data. The primary analyses compared, separately, the 12-week change in physical activity (mean PAL) and dietary intake (total energy) of the intervention and control groups using analysis of covariance (ANCOVA) models [42]. Baseline values of PAL and total energy intake were entered as covariates to control for chance imbalances at baseline, as well as the factors used in group allocation, i.e. sex, age and baseline BMI [43, 44]. Post hoc correlations were conducted to explore whether the intervention or control condition had different effects depending on participants’ age or baseline BMI, PAL or energy intake. Post hoc *t* tests were conducted for each condition to explore differences in effect depending on participant gender. ANCOVAs were also used for analysis of health outcome (anthropometrics and blood markers), controlling for baseline discrepancies in the dependent and allocation factors. *T* tests were conducted to assess whether the intervention had brought about change in the predicted cognitive determinants of behaviour as a manipulation check.

## Results

### Recruitment and Retention

**Fig. 1:**
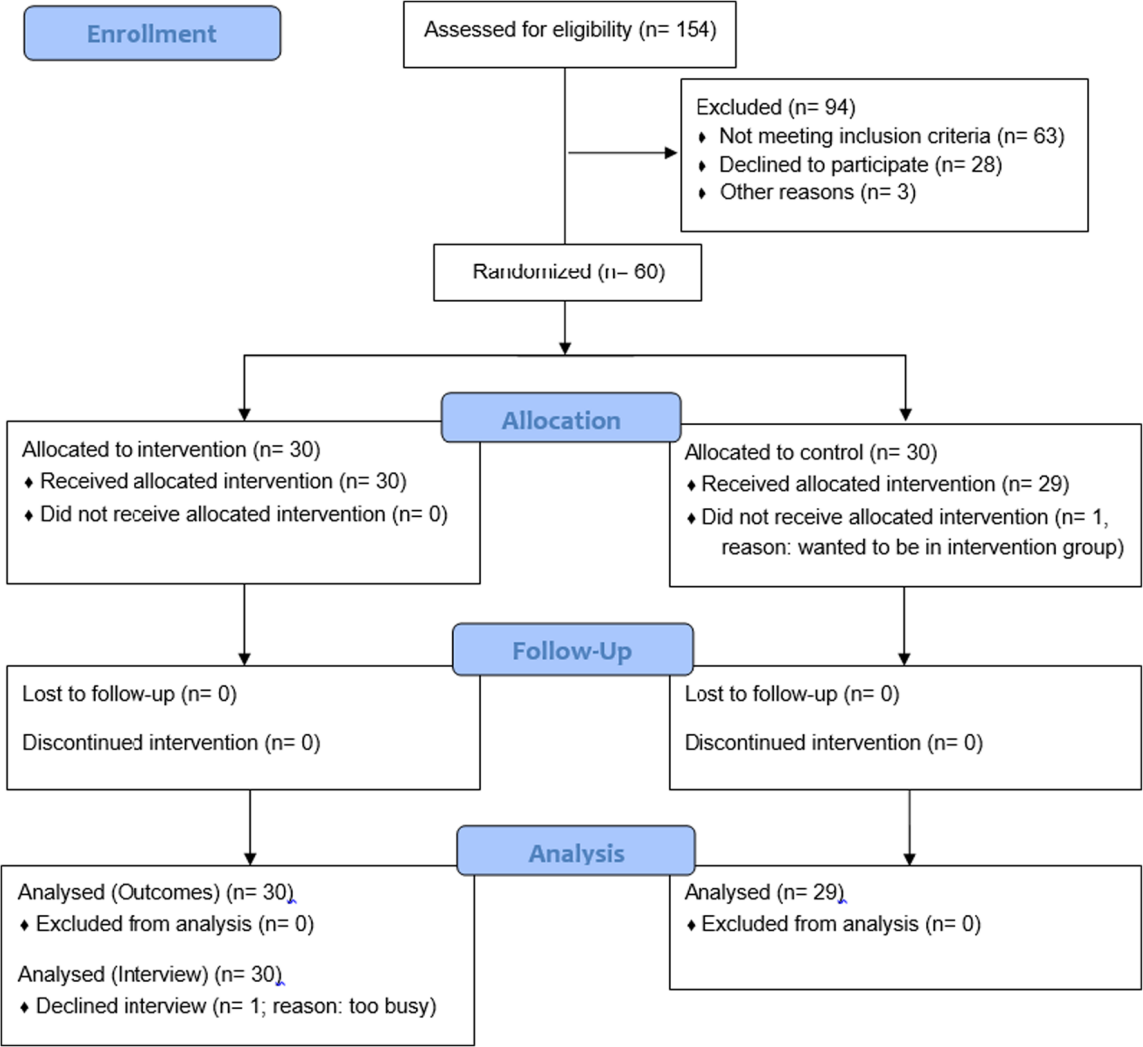
Participant flow diagram of Evolife trial

Figure 1 provides an overview of participant flow through the study, in line with CONSORT guidelines. Initial enquiries about participation were received from 154 people; 60 (38.9%) met the inclusion criteria and were allocated between the two groups from January to June 2017. After allocation, one participant dropped out of the control group before completing the baseline monitoring period—this participant was therefore excluded from analysis as no primary outcome data was collected from them. All other participants attended all three assessment visits and are included in the analysis. Total 24-h wear time of the activity monitors during the three assessment periods was good: on average, monitors were worn for between 94 and 97% of the time. One intervention group participant did not provide sufficient dietary intake data at 6 and 12 weeks. Dietary intake data were not received from three control group participants at 12 weeks. The trial ended in September 2017 when data had been collected from the last participant.

### Sample Characteristics

Baseline characteristics of the sample are displayed in Table 2; no significant differences were found between groups. Fifty-six percent of the sample was male and 95% white with a mean age of 50 years and mean BMI of 30.3 kg/m^2^. The mean index of multiple deprivation (IMD) of 7.61 indicates that the samples were reasonably affluent, living in areas considered to be in the 30–40% least deprived in England.

**Table 2:** Sample baseline characteristics

		Intervention (*N* = 30)	Control (*N* = 29)	*p* value
Sex, *N* (%)	Male	17 (57)	16 (55)	0.91
	Female	13 (43)	13 (45)	
Age, M (SD)		50.3 (8.9)	49.5 (9.1)	0.74
Age, min–max		35 - 70	35–68	
BMI, M (SD)		30.3 (4.2)	30.3 (3.0)	0.59
Race/ethnicity, *N* (%)	White	27 (90)	29 (100)	0.38
	Black/Black British	1 (3)	–	
	Asian/British Asian	1 (3)	–	
	Other	1 (3)	–	
Marital status, *N* (%)	Single	5 (17)	2 (7)	0.36
	Stable relationship	3 (10)	7 (24)	
	Married/civil partnership	20 (67)	19 (66)	
	Divorced/separated	2 (7)	1 (3)	
Employment, *N* (%)	Full time employment	22 (73)	23 (79)	0.74
	Part time employment	4 (13)	4 (14)	
	Student	1 (3)	–	
	Retired	2 (7)	2 (7)	
	Unemployed	1 (3)	–	
Education level, *N* (%)	Up to age 16 or less	2 (6)	3 (10)	0.47
	Up to age 18	2 (7)	5 (17)	
	Some additional	3 (10)	5 (17)	
	Undergraduate or higher degree	23 (77)	16 (56)	
IMD (1-10), M (SD)		7.9 (2.1)	7.3 (2.7)	0.23
Smoking, *N* (%)	Never smoked	18 (60)	13 (45)	0.15
	Ex-smoker	12 (40)	13 (45)	
	Currently smoke	–	3 (10)	
Immediate relations with CVD, *N* (%)	Yes	7 (23)	7 (24)	0.61
	No	22 (73)	22 (76)	
	Missing	1 (3)	–	
Immediate relations with T2DM, *N* (%)	Yes	6 (20)	7 (24)	0.70
	No	24 (80)	22 (76)	
Self-rating of health (0–100), M (SD)		63.7 (14.6)	66.7 (19.3)	0.40
Perceived mobility (1–3)		1.03 (0.18)	1.10 (0.31)	0.28
Perceived ability (1–3)		1.17 (0.46)	1.10 (0.31)	0.61
Perceived pain (1–3)		1.27 (0.45)	1.31 (0.47)	0.71
Perceived anxiety/depression (1–3)		1.33 (0.48)	1.28 (0.46)	0.63

*IMD* Index of Multiple Deprivation: 1 = most deprived 10%, 10 = least deprived 10%, *CVD* cardiovascular disease, *T2DM* type 2 diabetes mellitus

### Primary Outcomes—Physical Activity and Energy Intake at 12 Weeks

Physical activity and dietary outcomes are displayed in Table 3. The difference between groups’ 12-week change scores for PAL was of a small effect size but statistically non-significant (adjusted mean difference (AM∆) = 0.03, 95% CI = − 0.05 to 0.11, d = 0.32). The intervention group made a statistically significant increase of small effect size in PAL from baseline to 12 weeks (mean (standard deviation) 12-week change = 0.06 (0.15), t(28) = − 2.14, *p* < .05, d = 0.33), whereas the slight increase in PAL found for the control group did not reach significance (*p* > .05). No statistically significant differences between the control and intervention group were found for any of the other physical activity parameters at 12 weeks when adjusting for their baseline scores.

**Table 3:** Changes in physical activity and dietary intake

Variable	Group	Change 0–6 weeks (mean (SD))	AM∆^a^ (95% CI)	d (95% CI)	Change 0–12 weeks (mean (SD))	AM∆^a^ (95% CI)	d (95% CI)
*Physical activity*		*Int N = 30*, *Con N = 28*			*Int N = 29*, *Con N = 28*		
Daily PAL (TEE/BMR)	Int	0.04 (0.14)	− 0.001 (− 0.08, 0.08)	0.22 (− 0.30, 0.74)	0.06 (0.15)	0.03 (− 0.05, 0.11)	0.32 (− 0.20, 0.84)
	Con	< 0.01 (0.20)			0.01 (0.16)		
Sedentary (min/day)	Int	− 17 (61)	8 (− 26, 42)	− 0.07 (− 0.59, 0.44)	− 24 (58)	− 9 (− 39, 21)	− 0.25 (− 0.77, 0.27)
	Con	− 12 (89)			− 10 (59)		
Light (min/day)	Int	10 (34)	− 4 (− 21, 14)	0.05 (− 0.47, 0.56)	12 (33)	2 (− 15, 19)	0.11 (− 0.41, 0.63)
	Con	8 (42)			9 (29)		
MVPA (min/day)	Int	7 (40)	− 4 (− 26, 18)	0.07 (− 0.44, 0.59)	12 (44)	7 (− 17, 31)	0.24 (− 0.28, 0.76)
	Con	4 (60)			< 1 (50)		
TEE (kcal/day)	Int	37 (190)	5 (− 109, 119)	0.12 (− 0.40, 0.63)	67 (246)	80 (− 57, 216)	0.36 (− 0.17, 0.87)
	Con	9 (274)			− 23 (264)		
Daily steps	Int	1329 (3256)	830 (− 610, 2271)	0.35 (− 0.17, 0.86)	793 (3756)	560 (− 1145, 2265)	0.23 (− 0.29, 0.75)
	Con	401 (1785)			63 (2307)		
*Dietary intake*		*Int N=29*, *Con N=29*			*Int N=29*, *Con N=26*		
Total EI (kcal/day)	Int	− 330 (678)	− 198 (− 473, 76)	− 0.50 (− 1.01, 0.03)	− 431 (694)	− 214 (− 481, 53)	− 0.49 (− 1.02, 0.05)
	Con	− 28 (533)			− 124 (535)		
Total CHO (g/day)	Int	− 37.7 (82.0)	− 21.8 (− 53.5, 9.9)	− 0.49 (− 1.00, 0.04)	− 53.6 (85.1)	− 21.4 (− 54.3, 11.6)	− 0.46 (− 0.98, 0.09)
	Con	− 3.3 (55.9)			− 18.7 (65.7)		
Sugar (g/day)	Int	− 10.4 (29.8)	− 4.6 (− 16.8, 7.5)	− 0.34 (− 0.85, 0.19)	− 18.6 (29.4)	− 3.7 (− 19.0, 11.5)	− 0.24 (− 0.77, 0.30)
	Con	− 0.3 (30.1)			− 10.3 (39.9)		
Fibre (g/day)	Int	− 0.6 (10.4)	1.0 (− 3.1, 5.2)	0.08 (− 0.44, 0.59)	− 2.4 (8.7)	0.2 (− 3.3, 3.6)	− 0.03 (− 0.56, 0.50)
	Con	− 1.3 (7.8)			− 2.2 (7.1)		
Total fat (g/day)	Int	− 16.0 (33.4)	− 15.9 (− 29.2, − 2.6)	− 0.54 (− 1.06, − 0.01)	− 16.8 (34.9)	− 12.2 (− 24.7, 0.4)	− 0.38 (− 0.90, 0.16)
	Con	1.5 (30.9)			− 4.4 (31.5)		
Saturated fat (g/day)	Int	− 9.4 (13.7)	− 8.8 (− 15.0, − 2.7)	− 0.72 (− 1.24, − 0.17)	− 8.6 (15.6)	− 4.5 (− 9.8, 0.8)	− 0.36 (− 0.89, 0.18)
	Con	0.5 (13.9)			− 3.6 (12.0)		
Protein (g/day)	Int	− 7.7 (32.3)	− 8.5 (− 22.0, 4.9)	− 0.44 (− 0.96, 0.08)	− 11.8 (30.6)	− 3.4 (− 15.3, 8.5)	− 0.30 (− 0.82, 0.24)
	Con	5.2 (25.6)			− 3.7 (23.2)		
Sodium (mg/day)	Int	− 457.9 (1041.3)	− 136.2 (− 474.6, 202.3)	− 0.28 (− 0.79, 0.24)	− 500.9 (1091.1)	− 339.0 (− 736.9, 58.8)	− 0.39 (− 0.92, 0.15)
	Con	− 213.0 (685.7)			− 111.5 (887.6)		

At 6 weeks, intervention N = 30, control N = 28. At 12 weeks, intervention N = 29, control N = 28.

*AM*∆ adjusted mean difference, *Int* intervention group, *Con* control group, *CI* confidence interval, *PAL* physical activity level, *TEE* total energy expenditure, *BMR* basal metabolic rate, *MVPA* moderate to vigorous physical activity, *min/day* minutes per day, *EI* energy intake, *CHO* carbohydrate, *kcal/day* calories per day, *g/day* grams per day, *mg/day* milligrams per day

^a^Covariates in the model included sex, age, baseline BMI and baseline values of the dependent variable. For sedentary time and dietary variables, negative adjusted mean difference scores indicate a greater reduction was found for the intervention group

There was a statistically non-significant difference of small to medium effect size between groups’ 12-week change scores for total energy intake (AM∆ = − 214 kcal/day, 95% CI = − 481 to 53, d = − 0.49); the intervention group made a statistically significant reduction of large effect size in energy intake (mean (SD) 12-week change = − 431 kcal/day (694), t(28) = 3.34, *p* < 0.01, d = 0.81), whereas the slight decrease in energy intake found for the control group did not reach statistical significance (*p* > 0.05). In terms of dietary quality, the intervention group made a significantly greater reduction of small effect size in total fat intake by 12 weeks compared to the control group (AM∆ = − 12.15g/day, 95% CI = − 24.68, 0.37; F (1,49) = 3.8, *p* = 0.057; d = − 0.38). While there was a difference in the reduction of saturated fat intake of small effect size between groups at 12 weeks, the difference did not reach statistical significance (AM∆ = − 4.49 g/day, 95% CI = − 9.75, 0.77; F(1,49) = 2.94, *p* > 0.05; d = − 0.36). No other statistically significant differences between the two groups were found for the dietary parameters.

### Exploratory Subgroup Analysis of Intervention Effects

Results of post hoc exploratory correlations, to explore whether changes observed over the course of the study differed according to baseline characteristics, are shown in Table 4. For both groups, age was not associated with any of the primary outcome measures (12-week change scores for physical activity and dietary parameters). Baseline BMI was also not statistically significantly correlated with any of the primary physical activity outcomes for either group. In the intervention group, those with a higher baseline BMI made greater reductions in total energy intake and sugar intake at 12 weeks; in the control group, baseline BMI was not associated with any dietary outcome. Among the intervention group, baseline PAL was not associated with 12-week changes in any of the physical activity outcomes. Among the control group, those who were more active when they entered the study were less likely than those who were relatively inactive at baseline to increase their activity; the intervention condition had similar effects on physical activity regardless of baseline PAL. For both groups, people who consumed more at baseline were more likely to have made greater reductions in their dietary intake over the course of the study; this was shown to a greater extent among those in the intervention group. No significant differences in primary outcomes were found between males and females for either group.

**Table 4:** Correlations between baseline characteristics and primary outcome measures (12-week change scores for physical activity and dietary parameters) for each group

	Intervention				Control			
	Baseline PAL	Baseline EI	Age	Baseline BMI	Baseline PAL	Baseline EI	Age	Baseline BMI
Baseline PAL		0.13	0.05	− 0.22		0.10	− 0.13	− 0.02
Baseline EI			− 0.12	0.30			− 0.04	0.16
Age				− 0.36*				0.20
Daily PAL (TEE/BMR)	− 0.09		0.07	0.09	− 0.47*		− 0.24	− 0.21
Sedentary (min/day)	0.12		0.01	− 0.13	0.48**		0.18	0.08
Light (min/day)	− 0.07		− 0.08	0.12	− 0.07		− 0.08	0.03
Moderate (min/day)	− 0.16		0.02	0.09	− 0.52**		− 0.10	− 0.08
Vigorous (min/day)	0.03		0.07	0.03	− 0.21		− 0.27	− 0.08
MVPA (min/day)	− 0.11		0.05	0.08	− 0.52**		− 0.17	− 0.10
TEE (kcal)	− 0.21		0.02	0.16	− 0.55**		− 0.13	− 0.17
Daily steps	0.21		0.23	0.08	− 0.62**		− 0.06	− 0.17
Total EI (kcal/day)		− 0.77**	0.21	− 0.41*		− 0.43*	0.10	0.05
Total CHO (g/day)		− 0.59**	0.19	− 0.36		− 0.39*	0.74	− 0.13
Sugar (g/day)		− 0.45*	0.26	− 0.54**		− 0.01	− 0.01	− 0.14
Fibre (g/day)		− 0.32	0.03	− 0.30		− 0.13	0.26	0.10
Total fat (g/day)		− 0.71**	0.22	− 0.30		− 0.45*	0.05	0.06
Saturated fat (g/day)		− 0.75**	0.10	− 0.25		− 0.55*	− 0.16	− 0.10
Protein (g/day)		− 0.45*	0.24	− 0.36		− 0.18	0.30	0.11
Sodium (mg/day)		− 0.47**	− 0.02	− 0.01		− 0.26	0.06	0.12

N.B. A positive change score indicates an increase in the parameter from baseline to 12 weeks

*PAL* physical activity level, *TEE* total energy expenditure, *BMR* basal metabolic rate, *MVPA* moderate to vigorous physical activity, *min/day* minutes per day, *EI* energy intake, *CHO* carbohydrate, *kcal/day* calories per day, *g/day* grams per day

*p < 0.05; **p < 0.01

### Secondary Outcomes—Health Markers

There were no statistically significant differences between groups’ 12-week change scores for weight, BMI, waist circumference, blood pressure, or most of the blood biomarkers (see Table 5). A statistically significant difference between groups’ 12-week change in plasma glucose levels was found, indicating an increase in glucose for the control group (AM∆ = − 0.22 mmol/L, 95% CI = − 0.46 to 0.01, F (1,52) = 3.67, *p* < 0.05, d = − 0.36). In both groups, the reductions in systolic and diastolic blood pressure were of clinically meaningful magnitude.

**Table 5:** Changes in health markers

Variable	Group	Change 0–6 weeks (mean (SD))	AM∆ (95% CI)	Change 0–12 weeks (mean (SD))	AM∆ (95% CI)
Weight (kg)	Int	− 1.32 (2.40)	− 0.55 (− 1.58, 0.48)	− 2.03 (3.78)	− 0.62 (− 2.28, 1.05)
	Con	− 0.57 (1.49)		− 1.18 (2.16)	
BMI (kg/m^2^)	Int	− 0.41 (0.76)	− 0.22 (− 0.55, 0.10)	− 0.64 (1.18)	− 0.23 (− 0.74, 0.27)
	Con	− 0.18 (0.50)		− 0.4 (0.71)	
WC (cm)	Int	− 2.2 (3.2)	− 1.13 (− 2.76, 0.51)	− 2.9 (4.2)	− 1.31 (− 3.33, 0.71)
	Con	− 1.0 (3.4)		− 1.5 (3.7)	
Systolic BP (mmHg)	Int	− 3.7 (10.7)	− 2.49 (− 7.34, 2.36)	− 4.7 (9.7)	0.22 (− 4.21, 4.66)
	Con	− 0.8 (10.2)		− 4.4 (8.9)	
Diastolic BP (mmHg)	Int	− 2.0 (8.7)	0.03 (− 3.23, 3.29)	− 2.5 (6.4)	0.23 (− 2.37, 2.83)
	Con	− 2.5 (4.1)		− 3.2 (6.1)	
CRP (mg/L)	Int	− 0.02 (2.31)	− 0.30 (− 1.38, 0.78)	− 0.03 (1.75)	− 0.22 (− 0.98, 0.55)
	Con	0.23 (2.36)		0.22 (1.37)	
Triglycerides (mmol/L)	Int	− 0.11 (0.37)	− 0.15 (− 0.49, 0.18)	− 0.05 (0.39)	− 0.11 (− 0.41, 0.20)
	Con	0.02 (0.81)		0.06 (0.71)	
Total cholesterol (mmol/L)	Int	− 0.11 (0.60)	− 0.27 (− 0.56, 0.01)	− 0.02 (0.58)	− 0.24 (− 0.59, 0.11)
	Con	0.17 (0.55)		0.23 (0.76)	
HDL cholesterol (mmol/L)	Int	− 0.04 (0.28)	− 0.03 (− 0.14, 0.07)	− 0.06 (0.30)	− 0.05 (− 0.18, 0.07)
	Con	0.02 (0.12)		0.02 (0.26)	
LDL cholesterol (mmol/L)	Int	− 0.02 (0.47)	− 0.16 (− 0.41, 0.10)	0.06 (0.46)	− 0.12 (− 0.40, 0.16)
	Con	0.14 (0.54)		0.19 (0.60)	
Glucose (mmol/L)	Int	− 0.07 (0.52)	− 0.15 (− 0.40, 0.10)	0.02 (0.56)	− 0.22 (− 0.46, 0.01)
	Con	0.14 (0.48)		0.30 (0.44)	
Insulin (μU/L)	Int	− 0.86 (4.97)	− 0.51 (− 2.59, 1.57)	− 0.77 (5.35)	0.49 (− 1.47, 2.44)
	Con	0.17 (2.99)		− 0.61 (2.27)	
HOMA-IR	Int	− 0.21 (1.23)	− 0.26 (− 0.84, 0.32)	− 0.14 (1.17)	− 0.01 (− 0.50, 0.49)
	Con	0.16 (0.94)		− 0.01 (0.70)	

For systolic and diastolic BP, analyses excluded two participants (one in each group) who started new blood pressure controlling medication mid-study, thus Evolife *N* = 29 and control *N* = 28 for systolic and diastolic BP. For other anthropometric data, intervention *N* = 30, control *N* = 29 at all time points. For blood parameters (CRP to HOMA-IR), intervention *N* = 29, control *N* = 28. Healthy reference levels for blood markers: CRP < 1 mg/L, triglycerides < 1.7 mmol/L, total cholesterol < 5 mmol/L, LDL cholesterol < 3 mmol/L, HDL cholesterol > 1mmol/L, glucose ≤ 5.6 mmol/L

*kg* kilograms, *BMI* body mass index, *kg/m* ^*2*^ kilograms per metre squared, *WC* waist circumference, *cm* centimetres, *BP* blood pressure, *mmHg* millimetres of mercury, *mg/L* milligrams per litre, *mmol/L* millimoles per litre, *CRP* C-reactive protein, *μU/L* micro units per litre, *HOMA-IR* homeostasis model assessment for insulin resistance

### Cognitive Processes

Differences of small effect size that did not reach significance were found between groups’ 12-week change scores for autonomous motivation and self-efficacy for physical activity and autonomous motivation for consuming a healthy diet, indicating slightly greater increases for the intervention group (Physical activity: autonomous motivation AM∆ = 0.12, 95% CI = − 0.15 to 0.39, d = 0.27; self-efficacy AM∆ = 2.66, 95% CI = − 4.46 to 9.79, d = 0.22. Diet: autonomous motivation AM∆ = 0.23, 95% CI = − 0.07 to 0.53, d = 0.31). Significant differences of medium to large effect size were found between groups’ 12-week change scores self-monitoring of physical activity and action planning for both physical activity and diet, indicating greater increases for the intervention group (physical activity: self-monitoring AM∆ = 0.40, 95% CI = 0.09 to 0.71, d = 0.82; action planning AM∆ = 0.62, 95% CI = 0.19 to 1.04, d = 0.91. Diet: action planning AM∆ = 0.36, 95% CI = 0.02 to 0.70, d = 0.63). Relative to the control group, the intervention group also made greater increases, of small and medium effect size, in self-monitoring of diet (AM∆ = 0.15, 95% CI = − 0.13 to 0.43, d = 0.30) and coping planning for physical activity (AM∆ = 0.41, 95% CI = − 0.04 to 0.85, d = 0.65); however, the differences between groups for these two variables did not reach significance. There was no difference between groups in coping planning for diet.

## Discussion

The aim of this study was to evaluate the effectiveness of Evolife, a self-directed intervention that was designed to increase physical activity and reduce energy intake among overweight and obese adults. The intervention did not lead to significantly greater improvements in physical activity or reductions in energy intake compared with a minimal intervention control condition, involving a face-to-face introductory session to generic online resources and awareness that their health and health behaviours will be monitored. There was a trend for greater improvement in the intervention group and, in relation to other intervention studies, the effect sizes found for the relative change in behaviours are promising. For example, the changes in physical activity parameters were larger than the overall effect size reported by Davies and colleagues [45] in a systematic review for the effectiveness of Internet-delivered interventions on physical activity (d = 0.14). The effect sizes for between-group differences for both physical activity and dietary intake were similar to (or greater than) the overall effect size found in a meta-regression of cognitive-behavioural interventions for activity and healthy eating [20]. In terms of minutes spent active, the intervention produced a similar relative increase to that found in a systematic review of reviews, which included interventions targeting physical activity and/or diet, delivered face-to-face by trained providers (increase of 30–60 min per week; [21]). Given that the Evolife intervention was designed to be self-directed and encouraged participants to make small but sustainable changes to their lifestyles, the modest but meaningful behavioural outcomes are encouraging. However, the small sample size and lack of long-term follow-up mean that the results should be interpreted with caution.

Participants were able to choose their own dietary and physical activity goals; this flexible approach was adopted to increase and maintain participants’ engagement with the intervention (as they could choose goals that were relevant to their lifestyles). In addition, it was felt that any increase in physical activity would be beneficial and that aerobic, strengthening and flexibility-enhancing activities are all important for health. However, giving participants a more clearly defined type of activity to change (e.g. increase the number of minutes spent in moderate intensity activity) might have resulted in greater change in the outcomes (at least for the specified type of physical activity).

The study also examined whether any changes in activity or diet brought about by the 12-week intervention were sufficient to generate clinically meaningful changes in metabolic control and/or anthropometric risk markers for developing type 2 diabetes and cardiovascular disorder (although it should be noted that this study was not powered to detect such changes). There were no statistically significant differences between groups on these parameters. However, participants who received the intervention achieved an average weight loss of just over 2 kg by 12 weeks, which represents a clinically meaningful reduction sufficient to decrease an individual’s risk of cardiovascular disorders and type 2 diabetes [46]. Both groups made reductions in systolic and diastolic blood pressure of clinically meaningful magnitude (2 mmHg can significantly reduce the incidence of cardiovascular disorders in both hypertensive and normotensive individuals [47, 48]).

The intervention did lead to improvements in most of the intended cognitive determinants of behaviour; however, these may not have been of great enough magnitude to prompt behavioural change [49]. A full process evaluation and reporting of the interview findings is beyond the scope of this paper (these will be reported elsewhere) but, briefly, although the evolutionary mismatch concept was found to be interesting by participants and seemed to help make the health messages more meaningful for many, it was not sufficient to sustain engagement with the website. All participants reported visiting the website at least once a week, but the majority of participants viewed the information pages only once, shortly after the introductory session and then only revisited the goal setting page. Participants’ reports were partly corroborated by data from analytic software, showing that the website had been visited each week throughout the trial and that the most visited page was the goal setting one. Unfortunately, however, these data were not available at an individual level. Providing the informational content in phases or regularly updating the content might help to maintain engagement with the evolutionary message and in turn promote more and sustained motivation and self-efficacy for behaviour change.

This study was powered to detect a difference in PAL of medium effect size (d = 0.69), based on a similar 12-week intervention (24) involving an introductory session with a researcher, information about the effects of physical activity and health and an online programme to support goal setting and activity monitoring. The Evolife intervention targeted both physical activity and diet, and it might be that to have an expected change of medium effect size in both parameters over the same length of time was an overestimation, leading to a smaller-than-required sample size. A longer intervention or follow-up time might have delivered greater impact; however, a meta-analysis of 122 physical activity and dietary interventions (20) found that such design characteristics were not able to distinguish between effective and ineffective interventions.

A strength of this study was the use of objective measures of physical activity and health outcomes. For dietary intake, however, we relied on a self-reported measure (weighed food diaries) which might have been subject to under-reporting (39), although this is likely to have been the case for participants in both groups. It was obviously not possible to mask from participants the fact that their activity and diets were being monitored at the three time points, which could have led to participants changing their behaviour, consciously or unconsciously, as a result of knowing they were being observed. In an attempt to minimise this, the importance of continuing their normal behaviour (i.e. how they behaved in the weeks immediately prior to the monitoring period) was emphasised to participants both in terms of validity of the study and accuracy of the individual feedback that participants received at the end of the study. It should also be noted that the recruitment methods likely introduced a selection bias: by responding to advertisements for a health intervention study, participants demonstrated, to varying degrees, motivation to change their behaviour

## Conclusions

This exploratory randomised controlled trial tested the effectiveness of an evolutionary mismatch-framed, website-based intervention at promoting increases in physical activity and improvements in dietary intake among overweight and obese adults. The intervention did not lead to significantly greater improvement in physical activity or diet than a minimal intervention control condition. There was a trend for greater change in the intervention group and refinements to the intervention website might help to promote greater behaviour change; further research is needed to test whether the observed behavioural changes are maintained beyond the intervention period.
