# Correction to: Effects of a Web-Based, Evolutionary Mismatch-Framed Intervention Targeting Physical Activity and Diet: A Randomised Controlled Trial

**DOI:** 10.1007/s12529-023-10169-y

**Published:** 2023-03-07

**Authors:** Elisabeth B. Grey, Dylan Thompson, Fiona B. Gillison

**Affiliations:** 1 Centre for Motivation and Health Behaviour Change, Department for Health, University of Bath, Bath, BA2 7AY UK; 2 Department for Health, University of Bath, Bath, BA2 7AY UK

**Correction to: International Journal of Behavioral Medicine** **(2019) 26:645–657** 10.1007/s12529-019-09821-3

The authors have provided a link to the dataset, which was not available at the time of publication. The additional link is 10.15125/BATH-01245.

